# Effect of Acute Cold Exposure on Energy Metabolism and Activity of Brown Adipose Tissue in Humans: A Systematic Review and Meta-Analysis

**DOI:** 10.3389/fphys.2022.917084

**Published:** 2022-06-28

**Authors:** Chuanyi Huo, Zikai Song, Jianli Yin, Ying Zhu, Xiaohan Miao, Honghao Qian, Jia Wang, Lin Ye, Liting Zhou

**Affiliations:** ^1^ Department of Occupational and Environmental Health, School of Public Health, Jilin University, Changchun, China; ^2^ Department of Cardiology, the First Hospital of Jilin University, Changchun, China

**Keywords:** obesity, brown adipose tissue, hypothermia, energy expenditure, meta-analysis

## Abstract

**Background:** The benefit of cold exposure for humans against obesity has brought the energy metabolism and activity of brown adipose tissue (BAT) induced by cold into focus. But the results are inconsistent. This review is aimed to systematically explore the effect of cold exposure on the activity of BAT and energy metabolism in humans.

**Methods:** We searched relevant papers that were published from 1990 to 2021 and were cited in PubMed Central, Web of science, Embase and Cochrane Library databases to conduct this systematic review and meta-analysis. Energy metabolism, BAT volume, BAT activity and non-esterified fatty acids (NEFA) data reported in eligible researches were extracted. Meta-analysis was applied to combine the mean difference or standard mean difference with their 95% confidence intervals (95%CI). RevMan 5.3 software was used for meta-analysis and evaluating the risk of bias. Stata 16.0 was used for evaluating the publication bias.

**Results:** Ten randomized controlled trials were included in meta-analysis. Compared with human exposed in room temperature at 24°C, the energy expenditure (EE) was increased after acute cold exposure at 16∼19°C (Z = 7.58, *p* < 0.05, mean different = 188.43kal/d, 95% CI = 139.73–237.13); BAT volume (Z = 2.62, *p* < 0.05; standard mean different = 0.41, 95% CI = 0.10–0.73); BAT activity (Z = 2.05, *p* = 0.04, standard mean difference = 1.61, 95% CI = 0.07–3.14) and the intake of BAT NEFA (Z = 2.85, *p* < 0.05; standard mean different = 0.53, 95% CI = 0.17–0.90) also increased.

**Conclusion:** Acute cold exposure could improve the energy expenditure and BAT activity in adults, which is beneficial for human against obesity.

## Introduction

Nowadays, overweight and obesity have become the greatest health challenges worldwide. It was estimated that the percentage of overweight or obese adults was 39% for men and 40% for women; over 340 million children and adolescents aged 5–19 were overweight or obese in 2016. Moreover, World Health Organization (WHO) had pointed out that the epidemic of obesity is on the rise worldwide. Obesity affects nearly all physiological functions of the body and increases the risk of many diseases, such as diabetes mellitus, cardiovascular disease, musculoskeletal disorders and some cancers (including endometrial, breast, ovarian, kidney, and colon cancers) (Anandacoomarasamy et al., 2008; Singh et al., 2013; Lauby-Secretan et al., 2016).

Obesity is a state of energy imbalance, which means much more energy intake than energy expenditure, leading to excess energy stored as fat. Reducing energy intake and increasing energy consumption are effective ways to combat obesity. Brown adipose tissue (BAT), owing to its unique capacity to change excess energy into thermal energy, is considered an effective potential target against obesity and related metabolic diseases (Cypess and Kahn, 2010; Tseng et al., 2010). BAT is mainly regulated by the sympathetic nervous system (SNS) that releases norepinephrine to activate β-adrenergic receptor (β-AR) on the surface of brown adipocytes (Oelkrug et al., 2015). Subsequently, uncoupling protein 1 (UCP1) uses the proton gradient created by nicotinamide adenine dinucleotide (NADH) and flavin adenine dinucleotide (FADH2) instead of the decomposition of adenosine triphosphate (ATP) to generate heat (Oelkrug et al., 2015). When UCP1 is expressed in the BAT, it can invigorate the mitochondrial respiration to the most extreme sum in arrange to compensate for the gradient loss, in which process leading to energy consumption increasing (Cannon and Nedergaard, 2004). The BAT activity is higher in normal weight people than that in obese or overweight people. BAT activity is inversely correlated with body mass index (BMI), age and fat content in humans (Cypess et al., 2009; Yoneshiro et al., 2011b; Dinas et al., 2015).

At present, several mechanisms affecting the activity and metabolism of BAT, including diet, exercise, medicine and cold exposure, have been proved (Cannon and Nedergaard, 2004; Marlatt and Ravussin, 2017; Yau and Yen, 2020). Among them, cold exposure is the foremost well-studied method to activate BAT, as the primary role of BAT is to convert glucose and fat into heat by non-shivering thermogenesis (NST). Acute cold exposure (1–48 h) increased glucose uptake and improved insulin sensitivity; and free fatty acids (FFA) uptake and metabolism also increased in BAT(Ouellet et al., 2012; Leitner et al., 2017). Amid continuous cold stimulation, adipose tissue was remodeled to activate the thermogenic potential of both BAT and white adipose tissue (WAT). Incessant cold exposure causes metabolic changes within the BAT to maximize β-oxidation of fat acid from human cells and blood, electron transport action, and Ucp1 expression to produce heat (Cannon and Nedergaard, 2004; Blondin et al., 2014; Blondin et al., 2017a).

A few studies carried out in rodents had suggested that prolonged exposure to cold environment could activate the BAT further improve diet-induced obesity and its related complications, such as disturbed glucose and lipid homeostasis (Vallerand et al., 1986; Bartelt et al., 2011). Studies carried out in humans had also indicated that cold-induced BAT activation could enhance glucose uptake and improve whole-body glucose disposal and insulin sensitivity (Cypess et al., 2009; Saito et al., 2009; van Marken Lichtenbelt et al., 2009; Virtanen et al., 2009; Yoneshiro et al., 2013b; Blondin et al., 2015). After cold exposure, the resting metabolic rate in healthy people with detectable BAT levels increased by 14% (Chondronikola et al., 2014). However, studies carried out in aborigines living in the deserts of Australia and Bushmen population in the Kalahari Desert of southern Africa indicated that repeated nocturnal cold exposure did not increase the energy expenditure (EE) (Scholander et al., 1958; Wyndham and Morrison, 1958). Besides, no increase in EE was observed in obese individuals exposed to a short-term acute cold due to the small number of BAT activations (Hanssen et al., 2016). Similarly, sleeping in a room at 19°C for 1 month did not alter the cold-induced thermogenesis (CIT) (Lee et al., 2014). To date, due to the limited sample size of the population, the evidence of the effects of cold exposure on BAT activity and energy metabolism in humans is limited and inconsistent. Therefore, the present systematic review is carried out to assess the effect of acute cold exposure on human BAT activity and energy metabolism, and it is very crucial to provide accurate evidence to combat obesity and related metabolic diseases.

## Materials and Methods

### Search Strategy and Selection Criteria

We conducted the standard method according to the Preferred Reporting Items for Systematic Reviews and Meta-analysis (PRISMA) guidelines (Shamseer et al., 2015). Two independent authors (HCY and SZK) searched for related articles about the effects of cold exposure on BAT activity and EE in humans published from 1 January 1990 to 31 May 2021 in PubMed, Web of Science, Embase and Cochrane library databases. The search strategy in the PubMed database can be found in Appendix A. Following the different retrieval requirements of different databases, the connectives were appropriately adjusted. In addition, the reference lists of the included studies in systematic reviews searched out were searched to supplement the literature of this study that had not been initially searched out. The disagreement between two independent authors in the searching strategy was resolved by consensus.

### Inclusion and Exclusion Criteria

The articles were included or excluded by two independent authors (HCY and SZK), and the differences between the two authors were resolved through consensus or the third author (ZY). The studies we included were randomized controlled trials (RCT) carried out in humans with clear data of the sample size, the brown adipose tissue activity and energy metabolism before and after cold exposure. We excluded the studies with the influence of other intervention factors besides cold exposure, the studied carried out in animals, or lack the data of BAT activity and energy metabolism before and after cold exposure. For studies from the same research group, we only included the latest research report. Our research does not include reviews, editorials, letters, magazine articles and meeting minutes.

### Data Extraction and Quality Assessment

We imported all articles searched from various databases into EndNote and removed duplicates. Two authors (HCY and YJL) independently reviewed the title, abstract, and full text that met the inclusion criteria. For any discrepancies, the third author (ZY) will make the final decision. For the included studies, two authors (HCY and QHH) extracted the following information: 1) the name of the first author; 2) the year of publication; 3) the country; 4) the year(s) of study; 5) the type of study; 6) the number of participants; 7) the average age (if the mean was not available, using the median instead); 8) baseline data such as BMI. Cold exposure intervention data were also extracted. Finally, we extracted the main results data of cold exposure on BAT activity and energy metabolism measured by ^18^ F-Fluorodeoxyglucose (FDG), Positron Emission Tomography (PET), Computed Tomography (CT), Magnetic Resonance Imaging (MRI), and energy expenditure.

Two authors (HCY and QHH) independently conducted the risk of bias in the included studies that met the systematic review standards, and any differences in the risk of bias assessment were adjusted by the third author (ZY). For the qualified randomized controlled trials, we used the Cochrane Library bias assessment tool to assess the risk of bias (Higgins et al., 2011).

### Statistical Analysis

RevMan 5.3 and Stata 16.0 software were used to conduct the meta-analysis. Continuous, inverse variance were selected to summarize energy metabolism difference (kcal/d), BAT volume, BAT activity and non-esterified fatty acids (NEFA). The means and standard deviations were used to represent the difference in energy expenditure between participants who were exposed to cold environment and participants who did not expose to cold environment or were controlled, studies only provided subgroup means and standard deviations were analyzed after merging. Cochran’s Q test and *I* ^2^ statistics were used to assess the heterogeneity among studies, and *I* ^2^ > 50% indicates significant heterogeneity. Subgroup analysis was used for heterogeneity analysis. When *I* ^2^ ≤ 50%, the fixed-effect model was used for meta-analysis; when *I* ^2^ > 50%, the random effect model was used. We estimated the 95% confidence interval (CI) of the study and used the Z value and *p* value to test the statistical significance. When *p* < 0.05, it was considered to be statistically different. Where appropriate, we used the following formula to convert standard error (SE) into standard deviation (SD): SD = SE× √n .

## Results

### Literature Search and Selection

**FIGURE 1:**
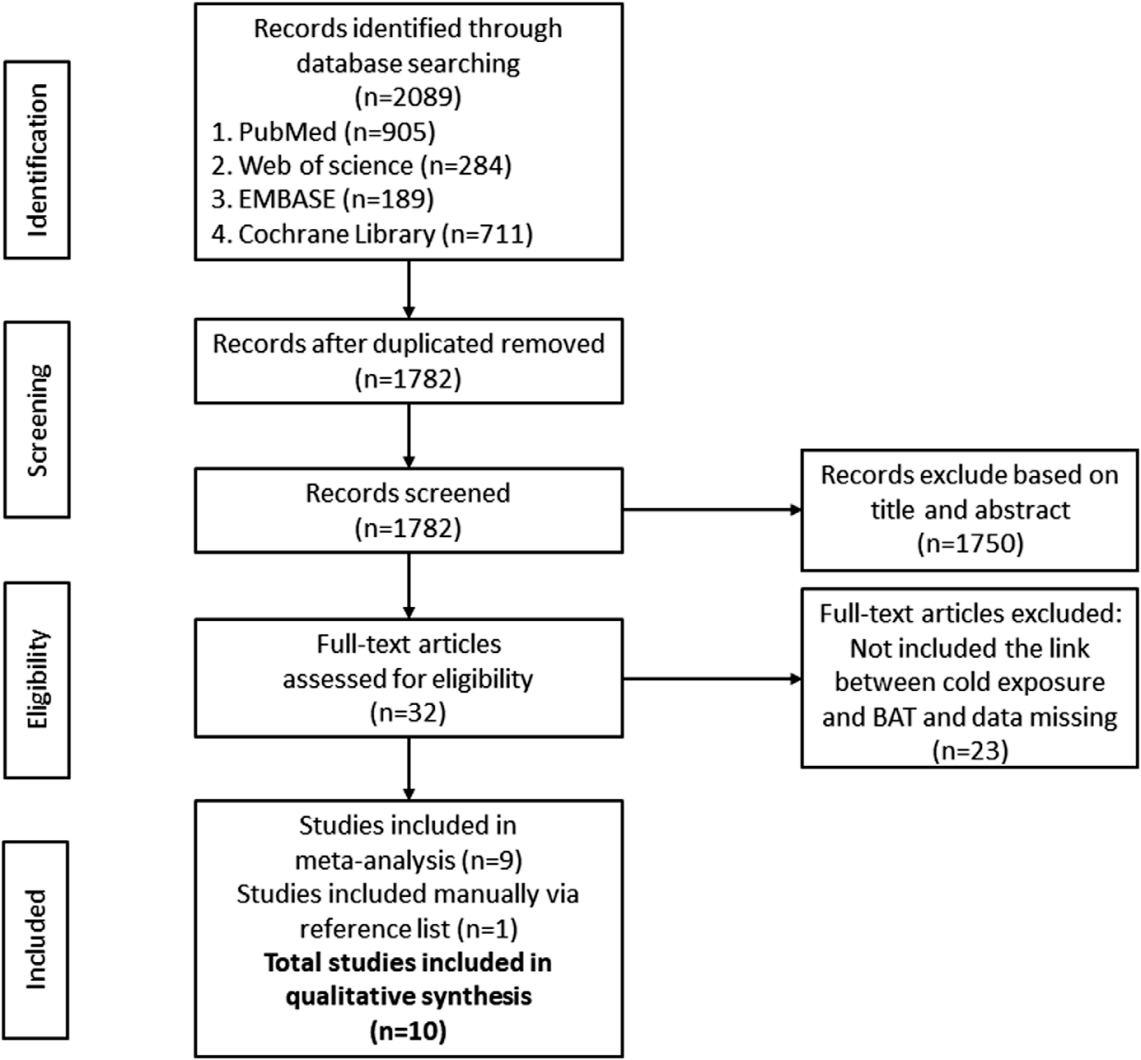
Flow Diagram of studies included in the systematic review and meta-analysis.

A total of 2089 documents were searched out initially. 307 duplicate articles, 1750 unrelated articles based on the review of the titles and abstracts were excluded. Of the remaining 32 articles, 23 were removed because some of them applied other interventions or did not clearly report EE, BAT volume, BAT activity or BAT NEFA. One study was manually included via searching the reference list. Finally, 10 RCT articles were included for meta-analysis. All of the 10 articles were in English. All participants in the 10 RCTs were healthy. After the qualification assessment, 10 papers reporting the BAT activity or energy expenditure after cold exposure were retained for quantitative analysis, 2 in Finland, 2 in the United States, 1 in Australia, 1 in Switzerland, 2 in the Netherlands and 2 in Japan. The research selection process and flowchart of the literature search were shown in Figure 1.

### Characteristics of the Included Studies

In general, the average age of participants was mainly 20∼40 years old, and the number of male participants was more than females. These studies all used FDG-PET/CT or MRI to evaluate the BAT activity before and after cold exposure, as well as the energy metabolism. Others baseline information can be found in Table 1.

**TABLE 1:** Characteristics of eligible studies.

Author	Year	Country	Design	Sample size	Age	BMI	Modality	Study design
Raiko JRH	2021	Finland	RCT	27 (M/F 13/24)	38.4	27.4	FDG-PET/CT	Cooled the temperature above their shivering point for 2 h
Taniguchi H	2020	Japan	RCT	6 (F)	21.3	20	FDG-PET/CT	Exposed to 19°C for 2 h
Brychta RJ	2019	United States	RCT	21 (L/O 13/24)	25.5	23.2 (L) 34.4 (O)	FDG-PET/CT	Exposed to 16°C for 4 h
Thuzar M	2018	Australia	RCT	13 (M/F 7/6)	28	24.0	FDG-PET/CT	Exposed to 19°C for 3 h
Romu T	2016	Sweden	RCT	25 (R/C 12/13)	25.2	22.5(R) 21.6(C)	MRI	Cooled the temperature above their shivering point for 1 h
Mueez UD	2016	Finland	RCT	7 (M/F 2/5)	36	25.5	FDG-PET/CT	Cooled the temperature above their shivering point for 2 h
Hanssen MJ	2015	Netherlands	RCT	16 (M/F 8/8)	21.3	21.3	FDG-PET/CT	Cooled the temperature above their shivering point for 30 min
Yoneshiro T	2013	Japan	RCT	8(M)	24.4	22.0	FDG-PET/CT	Exposed to 19°C for 2 h
Chen KY	2013	United States	RCT	24 (M/F 10/14)	28.1	--	FDG-PET	Exposed to19°C for 12 h
Van Marken Lichtenbelt	2009	Netherlands	RCT	24 (L/O 10/14)	--	23.2 (L) 30.3 (O)	FDG-PET/CT	Exposed to 16°C for 2 h

RCT, randomized controlled trial; M, males; F, females; L, lean; O, obese; R, room temperature; C, cold; BMI, body mass index; BAT NEFA, brown adipose tissue nonesterified fatty acid uptake; EE, energy expenditure.

### Risk of Bias Assessment Results

**FIGURE 2:**
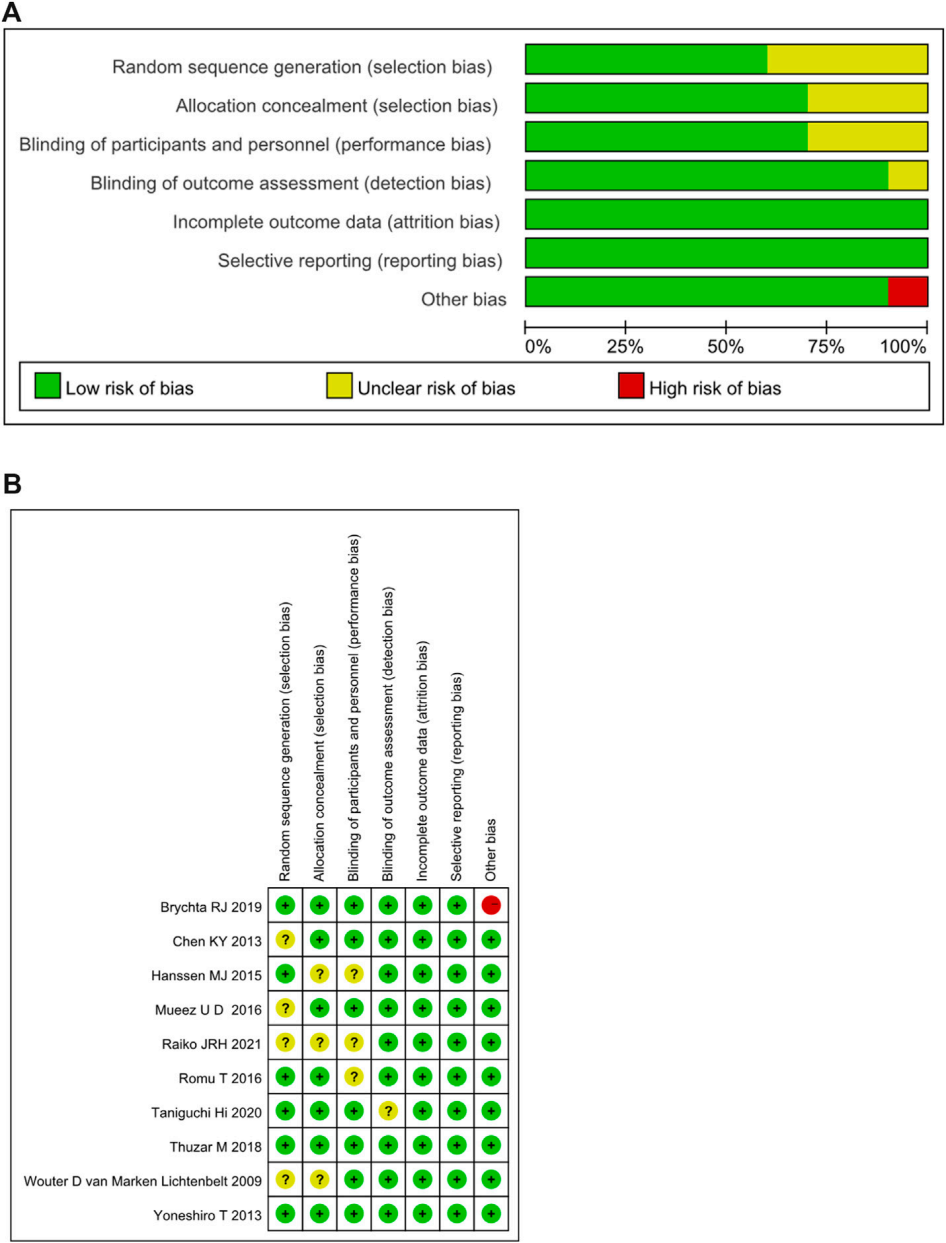
**(A)** Summary of risk of bias assessment for randomized controlled trials.**(B)** Risk of bias assessment for randomized controlled trials. **+**: low risk of bias; **-**: high risk of bias; ?: unclear risk of bias.

The bias risk assessment on the included RCTs was summarized in Figure 2A. 65% of the included RCTs showed a low risk in selection bias, and 35% showed an unclear risk of bias. In performance bias, 70% showed low risk, 30% showed an unclear risk of bias. In the detection bias, 90% showed a low risk, and 10% showed an unclear risk of bias. All of the included RCTs were low-risk in attrition bias and reporting bias. In the other bias, 90% displayed low risk, while 10% showed high risk. The detailed risk of bias for each study included in the systematic review was shown in Figure 2B.

### Meta-Analysis Outcomes

As for BAT volume, BAT activity and BAT NEFA, standard mean difference that can eliminate the influence of the absolute value of a study and the influence of measurement unit on the results was adopted. As for EE, mean difference was adopted.

**FIGURE 3:**
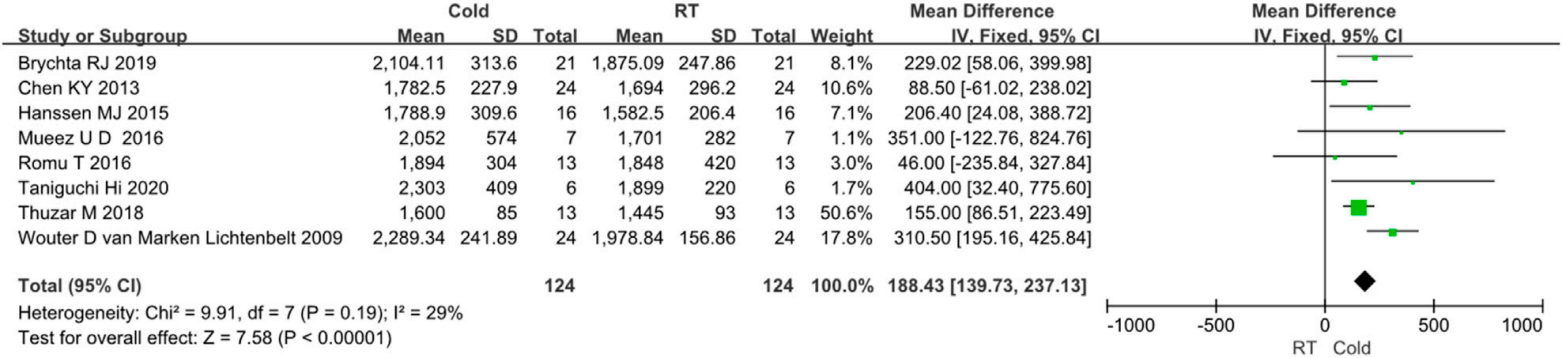
Forest plot for the effect of cold temperature on energy expenditure.

In the ten studies, eight studies reported clearly data of body energy expenditure before and after cold exposure (Taniguchi et al., 2020; Brychta et al., 2019; Thuzar et al., 2018; Romu et al., 2016; M et al., 2016; Hanssen MJ. et al., 2015; Chen et al., 2013; van Marken Lichtenbelt et al., 2009). Since *I* ^*2*^ < 50%, no obvious heterogeneity was observed. The results of the effect of cold exposure on body energy metabolism showed that the energy metabolism of subjects in the cold exposure group was significantly higher than that in the control group (*Z* = 7.58, *p* < 0.05, mean different = 188.43kal/d, *95% CI* = 139.73–237.13). The result was shown in Figure 3.

**FIGURE 4:**
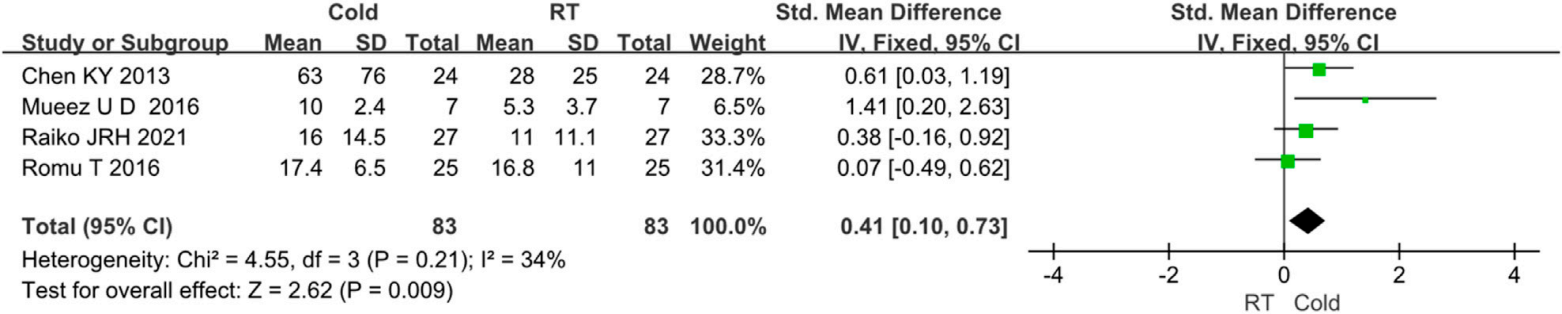
Forest plot for the effect of cold temperature on human brown adipose tissue volume.

The changes in BAT volume were evaluated in 4 studies (Raiko et al., 2021; Romu et al., 2016; M et al., 2016; Chen et al., 2013). Since *I* ^*2*^ < 50%, no obvious heterogeneity was observed. The results indicated that the volume of BAT was higher after cold exposure than that of room temperature (*Z* = 2.62, *p* < 0.05, standard mean difference = 0.41, 95% *CI* = 0.10–0.73). The result was shown in Figure 4.

**FIGURE 5:**
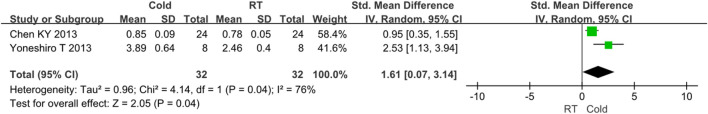
Forest plot for the effect of cold temperature on human brown adipose tissue activity.

As for BAT activity, two studies reported relevant data (Yoneshiro et al., 2013a; Chen et al., 2013). Because *I* ^*2*^ = 76% and *p* < 0.05, high heterogeneity was observed. Subgroup analysis was used for heterogeneity analysis. The results showed that the activity of BAT was improved after cold exposure (*Z* = 2.05, *p* = 0.04, standard mean difference = 1.61, 95% *CI* = 0.07–3.14). The result was shown in Figure 5.

**FIGURE 6:**
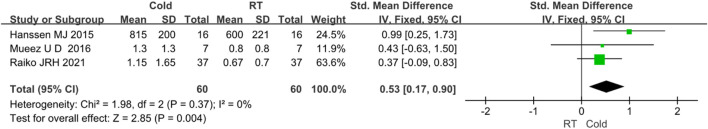
Forest plot for the effect of cold temperature on human brown adipose tissue NEFA uptake.

Three studies reported the intake of BAT NEFA (Raiko et al., 2021; M et al., 2016; Hanssen MJ. et al., 2015). Due to *I* ^*2*^ < 50%, no obvious heterogeneity was observed. The results showed that the intake of BAT NEFA was increased after cold exposure (*Z* = 2.85, *p* < 0.05, mean difference = 0.53, 95% *CI* = 0.17–0.90). The result was shown in Figure 6.

### Subgroups and Sensitivity Analysis

We performed sensitivity analyses via omitting 1 study each time. The analysis results indicated that the meta-analysis results for EE, BAT volume and BAT NEFA did not alter when each study was removed in turn, so that the findings were robust. The result was shown in Table 2.

**TABLE 2:** The pooled results of sensitivity analyses, (95% CI).

Indicators	Minimum estimate	Maximum estimate	Overall result
EE	161.95 (108.23–215.67)	222.62 (153.36–291.88)	188.43 (139.73–237.13)
BAT volume	0.34 (−0.03–0.70)	0.57 (0.20–0.95)	0.41 (0.10–0.73)
BAT NEFA	0.38 (−0.04–0.81)	0.81 (0.20–1.42)	0.53 (0.17–0.90)

EE, energy expenditure; BAT NEFA, brown adipose tissue nonesterified fatty acid uptake.

**FIGURE 7:**
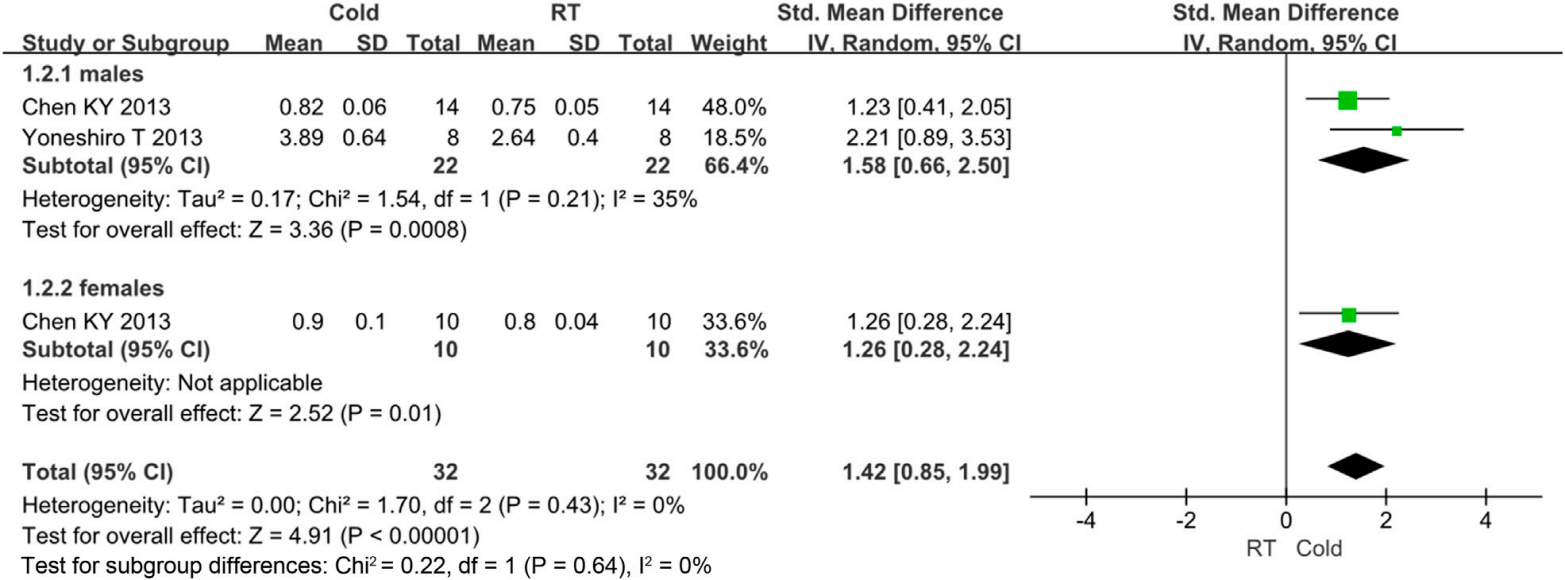
Forest plot for the brown adipose tissue activity of subgroup analysis.

There is some heterogeneity for the BAT activity, thus subgroup analysis is used to detect the heterogeneity. Only gender subgroup was analyzed. Other subgroups were not conducted because of under-representation in number of trials. The BAT activity was improved in males (standard mean difference = 1.58, 95% *CI* = 0.66–2.50) and females (standard mean difference = 1.26, 95% *CI* = 0.28–2.24). The result was shown in Figure 7.

### Publication Bias

**FIGURE 8:**
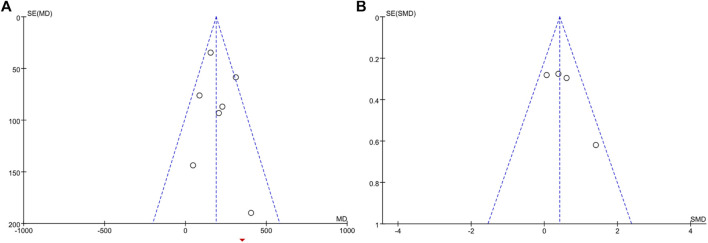
Funnel plots of RCTs recording energy expenditure **(A)** and BAT volume **(B)** outcomes.

Visual inspection of funnel plots revealed no asymmetry (Figure 8), and the results from Egger’s and Begg’s test indicated that no evidence for publication bias was detected for energy expenditure (Begg’s test, *p* = 0.386; Egger’s test, *p* = 0.521) and BAT volume (Begg’s test, *p* = 0.308; Egger’s test, *p* = 0.174).

## Discussion

Since BAT was “rediscovered” in humans, it has attracted great attention due to its potential ability to fight against obesity and related metabolic disorders (van Marken Lichtenbelt et al., 2009).

Although Wijers et al. reported that EE was significantly elevated in healthy lean subjects after cold exposure the present meta-analysis confirms that human energy expenditure is increased after acute cold exposure (van Marken Lichtenbelt, 2009; Wijers et al., 2010). Similarly, we found that the intake of NEFA is increased after acute cold exposure. Ouellet et al. also proved that plasma NEFA uptake is increased in cold-activated BAT compared with resting skeletal muscles and subcutaneous adipose tissues (Ouellet et al., 2012). Those evidences suggest that acute cold exposure plays an important role in enhancing energy metabolism. Even though the mechanism of cold-induced thermogenesis (CIT) is not fully defined, it has been previously indicated that glucose treatment, plasma glucose oxidation, and insulin sensitivity were improved effectively after acute cold exposure (Chondronikola et al., 2014). This mechanism is achieved by enhancing the expression of glucose transporter type 4 (GLUT4) or removing the triacylglycerol (TAG) in serum (Shibata et al., 1989; Shimizu et al., 1993; Baba et al., 2007; Bartelt et al., 2011; Nedergaard et al., 2011). Cold-induced increasing energy expenditure is related to the ^18^FDG positive BAT(Yoneshiro et al., 2011a; Vijgen et al., 2011). Whole body energy expenditure caused by cold in 18FDG BAT-positive subjects is greatly affected by seasonal changes (Yoneshiro et al., 2016). Free fatty acids as reaction substrates were used by BAT to generate heat (Cannon and Nedergaard, 2004). After cold exposure, NEFA from WAT lipolysis and lipoproteins (TRL) from triglyceride-rich lipoproteins enter BAT (Heeren and Scheja, 2018). Cold exposure promotes BAT activation to stimulate TAG lipolysis, then long-chain fatty acids were released and UCP1 was activated, leading to rodent and human mitochondrial thermogenesis (Bartelt et al., 2011; Nie et al., 2015; Blondin et al., 2017b). Acute cold exposure leads to an increase NEFA levels in plasma. And the genes related to lipid utilization are upregulated with cold exposure in humans. It is worth noting that activated BAT is correlated to cold-induced lipolysis, increased FFA re-esterification, FFA oxidation and energy expenditure compared to both lean and obese individuals with no or negligible BAT activity (Chondronikola et al., 2014; Blondin et al., 2015; Chondronikola et al., 2016). The reason for the difference in fatty acid intake in BAT may be related to the basal level of BAT and the low oxidative metabolism capacity in the activated state (Saari et al., 2020). However, during a 4-month mild cold exposure in healthy lean participants, BAT recruitability was confirmed by cold exposure, but significant CIT response augmentation did not be observed (Lee et al., 2014). This may be due to a slight increase in ambient temperature during the day, which damages BAT and blunts previous metabolic benefits. A 10-day cold acclimation period in patients with type 2 diabetes resulted in only a minor increase in metabolic activity of the supraclavicular BAT region (Hanssen M. J. W. et al., 2015). In addition to being overweight, these participants were older and already had low activity in this BAT region at baseline.

Moreover, our results show that both BAT activity and volume are improved after acute cold exposure. Our findings are agreement with the study of Hanssen et al., who reported obese subjects acquired large amounts of BAT during a short-term cold exposure periods (Hanssen et al., 2016). After cold exposure, oxygen consumption and blood perfusion in BAT are increased, which may be responsible for the increase of BAT volume. Although, the large number of specialized studies had investigated cold-activated BAT in humans, BAT volume is reported in few studies. Since a threshold based on PET SUV alone leads to an overestimation of BAT numbers, and a threshold based on SUV and CT HU leads to an underestimation of total BAT activity, different analyzing techniques need to be used to get BAT activity and BAT volume. FDG PET-CT is considered as a standard tool to identify human BAT, however, it has serious limitations that only tissues that actively ingest glucose can be detected (Cypess et al., 2014). However, instead of glucose, fatty acids were the main substrate of BAT function. As a result, bias may exist in the estimation of the quantification in BAT activity. Indeed, BAT activity is related to age, sex, environmental temperature and body fat content. A study showed that BAT was detected more than 50% in subjects aged 20–29 years, and less than 10% aged 50 and older (Yoneshiro et al., 2011b). In addition, some studies have found that PET/CT studies have observed a higher prevalence of BAT in winter than in summer, possibly due to the changes in environmental temperature (Cypess et al., 2009; Saito et al., 2009; Lee et al., 2010; Ouellet et al., 2011). BAT activity is negatively correlated with BMI, and the activity of healthy people is greater than that of overweight or obese people. The reason for this fact was that the supraclavicular fat depot in obese people is dominated by white fat, which leads to the weakening of BAT activation ability (van Marken Lichtenbelt et al., 2009).

The effects of cold exposure and BAT on energy metabolism have been intensively studied in animal models, but some points should be noted when we apply these studies to humans. Firstly, the fat depots in mice mainly located in the interscapular region and in the cervical spine, around the heart and kidneys, while in adult humans they mainly located in cervical, paravertebral, axillary and clavicular regions (Townsend and Tseng, 2015; Ikeda et al., 2018). Secondly, studies in rodents proved two type of BAT, “classical and brite”, which differ in development origin (Petrovic et al., 2010; Wu et al., 2012). Brown adipocytes, located in the interscapular region, originate from Myf5-positive myoblastic cells that called classical BAT(Timmons et al., 2007; Seale et al., 2008). Brite cells, named “beige or brite adipocytes”, originate from a Myf5-negative precursor cells, and/or the formation of brite cells within the white adipose depot is referred to as “browning” (Walden et al., 2012; Wu et al., 2012). BAT depots around the neck and in the supraclavicular regions of humans may comprise both classical brown and brite/beige cells, but its distribution may be affected by age and region (Jespersen et al., 2013; Lidell et al., 2013). Therefore, further studies are needed to distinguish the types of human BAT more clearly, to elucidate their role and mechanism in whole-body energy. When exposure to low temperature for a long time, it can promote browning of white adipose tissue, and increases the amount of brown adipocytes and the number of UCP1 through proliferation of interstitial preadipocytes and matured adipocytes of animals (Bukowiecki et al., 1986; Okamatsu-Ogura et al., 2017). Although experiments in rodents have shown the profound effects of cold on process of browning, the results of human experiments remain inconclusive. Studies in healthy human scWAT cells suggested that long-term cold exposure increased UCP1 expression and mitochondrial activity, which are characteristic of beige cells (Kern et al., 2014; Finlin et al., 2017). But a cold-adaptation study in healthy humans did not show scWAT beiging (van der Lans et al., 2013). Besides, 2–6 weeks cold exposure increases the volume of BAT, but it is not possible whether the increase volume was from enhanced activity or from an expanded cell mass (Blondin et al., 2014; Lee et al., 2014). While long-term cold exposure can promote beiging in cells, further research is needed to determine the temperature and time to induce beiging in humans, and the feasibility of these results applying. Furthermore, Compared with rodents, human have relatively less BAT, with 0.02% of body weight in humans and 0.4–1% of body weight in rodents (Geisler, 2011). Besides, mice are often housed at 22°C, a temperature different from the thermoneutrality (29–30°C) (de Jong et al., 2019). Such adaptation renders their BAT chronically activated, which may influence their energy metabolic behavior. Humans can protect themselves against a cold challenge, leading to BAT at a lower level. This may explain the differences in BAT activity and energy metabolism caused by cold exposure in animals and humans.

The current meta-analysis presents a response to the view that acute cold exposure to BAT is beneficial to human health, and there is also an evidence that chronic cold exposure can confer benefits on metabolic health. For example, sustained 6-week cold exposure resulted in increased BAT activity and CIT, along with decreased body fat mass (Yoneshiro et al., 2013a). Although, the potential of BAT activation to increase energy consumption inducing weight loss is exciting. Unfortunately, BAT activation through CIT in humans has just increased the energy expenditure and decreased the fat mass without eliciting any loss in body weight (Yoneshiro et al., 2013b). A study showed that BAT prevalence increased as an acute response to cold environment (less than 7 days), and the effect was neutralized with delayed of low temperatures (Kim et al., 2008). Moreover, maximal activation of BAT for extended periods is a difficult feat, particularly in humans. Compared with rodents, exposure humans to a severe and delayed environment is impractical. On the other hand, prolonged cold stimulation leads to a compensatory increase in people’s food intake, which to some extent counteracts the effect of BAT activation against obesity (Ravussin et al., 2014). Therefore, chronic cold exposure may cannot promote weight loss. Conversely, acute cold exposure holds a great potential for combating with obesity due to its ease of implementation. In the future, combining acute cold exposure with other modalities such as exercise, diet, and medication could be considered as a meaningful contribution to addressing obesity and metabolic disorder.

## Conclusion

BAT activity and volume had been increased after acute cold exposure. BAT also played an important role in regulating metabolism. After cold exposure, the body’s energy metabolic increases as well as NEFA intake, both of which indicated that it could regulate metabolism and increase heat production. BAT thermogenesis may be available as therapies to against obesity in the near future.

## Data Availability

The original contributions presented in the study are included in the article/supplementary material, further inquiries can be directed to the corresponding authors.

## Fundings

This work was sponsored by the Science and Technology Projects of Jilin provincial science and Technoogy Department (20200201523JC) and the Education Department of Jilin Province (JJKH20201052KJ).

## Abbreviations

WHO, world health organization; BAT, brown adipose tissue; SNS, sympathetic nervous system; β-AR, β-adrenergic receptor; UCP1, uncoupling protein one; NADH, nicotinamide adenine dinucleotide; FADH2, flavin adenine dinucleotide; ATP, adenosine triphosphate; BMI, body mass index; NST, non-shivering thermogenesis; FFA, free fatty acids; WAT, white adipose tissue; EE, energy expenditure; CIT, cold-induced thermogenesis; RCT, randomized controlled trials; FDG, ^18^ F-fluorodeoxyglucose; PET, positron emission tomography; MRI, magnetic resonance imaging; CT, computed tomography; NEFA, non-esterified fatty acids; CI, confidence interval; SE, standard error; SD, standard deviation; GLUT4, glucose transporter type 4; TAG, removing triacylglycerol; TRL, lipolysis and lipoproteins.

## Appendix ASearch Algorithm in PubMed

{[“brown adipose tissue” (Title/Abstract) OR “brown fat” (Title/Abstract) OR “brown like adipose tissue” (Title/Abstract) OR “beige” (Title/Abstract) OR “brown like fat” (Title/Abstract) OR “brown adipose like phenotype” (Title/Abstract) OR “browning process” (Title/Abstract) AND “hypothermia” (Title/Abstract)] OR “hypothermia induced” [ Title/Abstract] OR “induced mild hypothermia” [Title/Abstract]} AND 1990/01/01:2021/5/31 [Date-Publication].
